# Supplementary material for: Pyruvate Dehydrogenase Kinase 1 inhibition mediated oxidative phosphorylation enhancement in cartilage promotes osteoarthritis progression
Source: BMC Musculoskelet Disord. 2023 Jul 20;24:597. doi: 10.1186/s12891-023-06585-6 (PMC10357736; doi:10.1186/s12891-023-06585-6)
Supplement: Supplementary file 1 — Additional file 1. [file 12891_2023_6585_MOESM1_ESM.zip › BP.Gsea.1653623667859/GOBP_RIBOSOME_ASSEMBLY.html]

Details for gene set GOBP\_RIBOSOME\_ASSEMBLY[GSEA]

|  || Dataset | 55457\_collapsed\_to\_symbols.cls.cls #OA\_versus\_normal.cls.cls #OA\_versus\_normal\_repos |
| Phenotype | cls.cls#OA\_versus\_normal\_repos |
| Upregulated in class | normal |
| GeneSet | GOBP\_RIBOSOME\_ASSEMBLY |
| Enrichment Score (ES) | -0.5090209 |
| Normalized Enrichment Score (NES) | -1.943003 |
| Nominal p-value | 0.0 |
| FDR q-value | 0.04185447 |
| FWER p-Value | 0.512 |
Table: GSEA Results Summary

  

Fig 1: Enrichment plot: GOBP\_RIBOSOME\_ASSEMBLY      
 Profile of the Running ES Score & Positions of GeneSet Members on the Rank Ordered List

  

| SYMBOL | TITLE | RANK IN GENE LIST | RANK METRIC SCORE | RUNNING ES | CORE ENRICHMENT || 1 | NGRN | "neugrin, neurite outgrowth associated [Source:HGNC Symbol;Acc:HGNC:18077]" | 741 | 0.583 | -0.0288 | No |
| 2 | DHX29 | DExH-box helicase 29 [Source:HGNC Symbol;Acc:HGNC:15815] | 1012 | 0.515 | -0.0244 | No |
| 3 | XRCC5 | X-ray repair cross complementing 5 [Source:HGNC Symbol;Acc:HGNC:12833] | 1018 | 0.513 | 0.0006 | No |
| 4 | MRPS11 | mitochondrial ribosomal protein S11 [Source:HGNC Symbol;Acc:HGNC:14050] | 1305 | 0.457 | 0.0009 | No |
| 5 | MRPL22 | mitochondrial ribosomal protein L22 [Source:HGNC Symbol;Acc:HGNC:14480] | 1311 | 0.456 | 0.0231 | No |
| 6 | MRPS7 | mitochondrial ribosomal protein S7 [Source:HGNC Symbol;Acc:HGNC:14499] | 1650 | 0.406 | 0.0169 | No |
| 7 | EFL1 | elongation factor like GTPase 1 [Source:HGNC Symbol;Acc:HGNC:25789] | 2501 | 0.303 | -0.0343 | No |
| 8 | C1QBP | complement C1q binding protein [Source:HGNC Symbol;Acc:HGNC:1243] | 3083 | 0.241 | -0.0676 | No |
| 9 | RPS27L | ribosomal protein S27 like [Source:HGNC Symbol;Acc:HGNC:18476] | 3882 | 0.166 | -0.1215 | No |
| 10 | DDX28 | DEAD-box helicase 28 [Source:HGNC Symbol;Acc:HGNC:17330] | 4602 | 0.105 | -0.1723 | No |
| 11 | ABT1 | activator of basal transcription 1 [Source:HGNC Symbol;Acc:HGNC:17369] | 4783 | 0.088 | -0.1819 | No |
| 12 | RPS19 | ribosomal protein S19 [Source:HGNC Symbol;Acc:HGNC:10402] | 5952 | -0.002 | -0.2727 | No |
| 13 | MRPL20 | mitochondrial ribosomal protein L20 [Source:HGNC Symbol;Acc:HGNC:14478] | 5989 | -0.004 | -0.2754 | No |
| 14 | EIF6 | eukaryotic translation initiation factor 6 [Source:HGNC Symbol;Acc:HGNC:6159] | 6140 | -0.015 | -0.2863 | No |
| 15 | ERAL1 | Era like 12S mitochondrial rRNA chaperone 1 [Source:HGNC Symbol;Acc:HGNC:3424] | 6158 | -0.017 | -0.2868 | No |
| 16 | NOP53 | NOP53 ribosome biogenesis factor [Source:HGNC Symbol;Acc:HGNC:4333] | 6334 | -0.031 | -0.2989 | No |
| 17 | RPL10L | ribosomal protein L10 like [Source:HGNC Symbol;Acc:HGNC:17976] | 6502 | -0.042 | -0.3098 | No |
| 18 | MTERF4 | mitochondrial transcription termination factor 4 [Source:HGNC Symbol;Acc:HGNC:28785] | 6972 | -0.078 | -0.3424 | No |
| 19 | NLE1 | notchless homolog 1 [Source:HGNC Symbol;Acc:HGNC:19889] | 7253 | -0.099 | -0.3593 | No |
| 20 | FASTKD2 | FAST kinase domains 2 [Source:HGNC Symbol;Acc:HGNC:29160] | 7730 | -0.136 | -0.3896 | No |
| 21 | MRPS2 | mitochondrial ribosomal protein S2 [Source:HGNC Symbol;Acc:HGNC:14495] | 7863 | -0.146 | -0.3927 | No |
| 22 | RPL3L | ribosomal protein L3 like [Source:HGNC Symbol;Acc:HGNC:10351] | 8097 | -0.163 | -0.4028 | No |
| 23 | DHX30 | DExH-box helicase 30 [Source:HGNC Symbol;Acc:HGNC:16716] | 8111 | -0.164 | -0.3957 | No |
| 24 | MTERF3 | mitochondrial transcription termination factor 3 [Source:HGNC Symbol;Acc:HGNC:24258] | 8144 | -0.166 | -0.3900 | No |
| 25 | RPSA | ribosomal protein SA [Source:HGNC Symbol;Acc:HGNC:6502] | 8866 | -0.222 | -0.4351 | No |
| 26 | PRKDC | "protein kinase, DNA-activated, catalytic subunit [Source:HGNC Symbol;Acc:HGNC:9413]" | 9632 | -0.286 | -0.4805 | No |
| 27 | NIP7 | nucleolar pre-rRNA processing protein NIP7 [Source:HGNC Symbol;Acc:HGNC:24328] | 9950 | -0.316 | -0.4896 | No |
| 28 | RPL5 | ribosomal protein L5 [Source:HGNC Symbol;Acc:HGNC:10360] | 10201 | -0.341 | -0.4922 | Yes |
| 29 | RPL24 | ribosomal protein L24 [Source:HGNC Symbol;Acc:HGNC:10325] | 10263 | -0.347 | -0.4797 | Yes |
| 30 | BRIX1 | biogenesis of ribosomes BRX1 [Source:HGNC Symbol;Acc:HGNC:24170] | 10275 | -0.348 | -0.4634 | Yes |
| 31 | MRTO4 | "MRT4 homolog, ribosome maturation factor [Source:HGNC Symbol;Acc:HGNC:18477]" | 10300 | -0.350 | -0.4479 | Yes |
| 32 | RRP7A | ribosomal RNA processing 7 homolog A [Source:HGNC Symbol;Acc:HGNC:24286] | 10449 | -0.367 | -0.4413 | Yes |
| 33 | RPS14 | ribosomal protein S14 [Source:HGNC Symbol;Acc:HGNC:10387] | 10558 | -0.379 | -0.4310 | Yes |
| 34 | RPL10 | ribosomal protein L10 [Source:HGNC Symbol;Acc:HGNC:10298] | 10684 | -0.391 | -0.4213 | Yes |
| 35 | RRS1 | ribosome biogenesis regulator 1 homolog [Source:HGNC Symbol;Acc:HGNC:17083] | 11045 | -0.437 | -0.4278 | Yes |
| 36 | RPL38 | ribosomal protein L38 [Source:HGNC Symbol;Acc:HGNC:10349] | 11122 | -0.446 | -0.4116 | Yes |
| 37 | RPL6 | ribosomal protein L6 [Source:HGNC Symbol;Acc:HGNC:10362] | 11129 | -0.447 | -0.3900 | Yes |
| 38 | RPS5 | ribosomal protein S5 [Source:HGNC Symbol;Acc:HGNC:10426] | 11206 | -0.458 | -0.3733 | Yes |
| 39 | RPL11 | ribosomal protein L11 [Source:HGNC Symbol;Acc:HGNC:10301] | 11252 | -0.464 | -0.3538 | Yes |
| 40 | PWP2 | PWP2 small subunit processome component [Source:HGNC Symbol;Acc:HGNC:9711] | 11604 | -0.516 | -0.3556 | Yes |
| 41 | RPS15 | ribosomal protein S15 [Source:HGNC Symbol;Acc:HGNC:10388] | 11671 | -0.527 | -0.3347 | Yes |
| 42 | MDN1 | midasin AAA ATPase 1 [Source:HGNC Symbol;Acc:HGNC:18302] | 11973 | -0.588 | -0.3290 | Yes |
| 43 | PPAN | peter pan homolog [Source:HGNC Symbol;Acc:HGNC:9227] | 12022 | -0.597 | -0.3032 | Yes |
| 44 | RPS27 | ribosomal protein S27 [Source:HGNC Symbol;Acc:HGNC:10416] | 12071 | -0.612 | -0.2767 | Yes |
| 45 | VCX | variable charge X-linked [Source:HGNC Symbol;Acc:HGNC:12667] | 12110 | -0.623 | -0.2488 | Yes |
| 46 | RPLP0 | ribosomal protein lateral stalk subunit P0 [Source:HGNC Symbol;Acc:HGNC:10371] | 12285 | -0.672 | -0.2292 | Yes |
| 47 | RPL3 | ribosomal protein L3 [Source:HGNC Symbol;Acc:HGNC:10332] | 12350 | -0.691 | -0.2000 | Yes |
| 48 | NOP2 | NOP2 nucleolar protein [Source:HGNC Symbol;Acc:HGNC:7867] | 12372 | -0.699 | -0.1670 | Yes |
| 49 | RPS28 | ribosomal protein S28 [Source:HGNC Symbol;Acc:HGNC:10418] | 12526 | -0.761 | -0.1413 | Yes |
| 50 | NPM1 | nucleophosmin 1 [Source:HGNC Symbol;Acc:HGNC:7910] | 12530 | -0.762 | -0.1039 | Yes |
| 51 | BOP1 | BOP1 ribosomal biogenesis factor [Source:HGNC Symbol;Acc:HGNC:15519] | 12580 | -0.785 | -0.0689 | Yes |
| 52 | RPL23A | ribosomal protein L23a [Source:HGNC Symbol;Acc:HGNC:10317] | 12785 | -0.944 | -0.0381 | Yes |
| 53 | DDX3X | DEAD-box helicase 3 X-linked [Source:HGNC Symbol;Acc:HGNC:2745] | 12788 | -0.949 | 0.0087 | Yes |
Table: GSEA details [plain text format]

  

Fig 2: GOBP\_RIBOSOME\_ASSEMBLY      
 Blue-Pink O' Gram in the Space of the Analyzed GeneSet

  

Fig 3: GOBP\_RIBOSOME\_ASSEMBLY: Random ES distribution      
 Gene set null distribution of ES for **GOBP\_RIBOSOME\_ASSEMBLY**
